# Estimation of scrub typhus incidence and spatiotemporal multicomponent characteristics from 2016 to 2023 in Zhejiang Province, China

**DOI:** 10.3389/fpubh.2024.1359318

**Published:** 2024-09-26

**Authors:** Haocheng Wu, Ming Xue, Chen Wu, Qinbao Lu, Zheyuan Ding, Xinyi Wang, Tianyin Fu, Ke Yang, Junfen Lin

**Affiliations:** ^1^Zhejiang Provincial Center for Disease Control and Prevention, Hangzhou, Zhejiang, China; ^2^Zhejiang Key Lab of Vaccine, Infectious Disease Prevention and Control, Hangzhou, Zhejiang, China; ^3^Hangzhou Centre for Disease Control and Prevention, Hangzhou, Zhejiang, China

**Keywords:** scrub typhus, multivariate time series model, kriging interpolation, wavelet analysis, random effect

## Abstract

**Background:**

China is one of the main epidemic areas of scrub typhus, and Zhejiang Province, which is located in the coastal area of southeastern China, is considered a key region of scrub typhus. However, there may be significant bias in the number of reported cases of scrub typhus, to the extent that its epidemiological patterns are not clearly understood. The purpose of this study was to estimate the possible incidence of scrub typhus and to identify the main driving components affecting the occurrence of scrub typhus at the county level.

**Methods:**

Data on patients with scrub typhus diagnosed at medical institutions between January 2016 and December 2023 were collected from the China Disease Control and Prevention Information System (CDCPIS). The kriging interpolation method was used to estimate the possible incidence of scrub typhus. Additionally, a multivariate time series model was applied to identify the main driving components affecting the occurrence of scrub typhus in different regions.

**Results:**

From January 2016 to September 2023, 2,678 cases of scrub typhus were reported in Zhejiang Province, including 1 case of reported death, with an overall case fatality rate of 0.04%. The seasonal characteristics of scrub typhus in Zhejiang Province followed an annual single peak model, and the months of peak onset in different cities were different. The estimated area with case occurrence was relatively wider. There were 41 counties in Zhejiang Province with an annual reported case count of less than 1, while from the estimated annual incidence, the number of counties with less than 1 case decreased to 21. The average annual number of cases in most regions fluctuated between 0 and 15. The numbers of cases in the central urban area of Hangzhou city, Jiaxin city and Huzhou city did not exceed 5. The estimated random effect variance parameters $\sigma_{\lambda }^{2}$ , $\sigma_{\phi }^{2}$ , and $\sigma_{\nu }^{2}$ were 0.48, 1.03 and 3.48, respectively. The endemic component values of the top 10 counties were Shuichang, Cangnan, Chun’an, Xinchang, Pingyang, Xianju, Longquan, Dongyang, Yueqing and Qingyuan. The spatiotemporal component values of the top 10 counties were Pujiang, Anji, Pan’an, Dongyang, Jinyun, Ninghai, Yongjia, Xiaoshan, Yinwu and Shengzhou. The autoregressive component values of the top 10 counties were Lin’an, Cangnan, Chun’an, Yiwu, Pujiang, Longquan, Xinchang, Luqiao, Sanmen and Fuyang.

**Conclusion:**

The estimated incidence was higher than the current reported number of cases, and the possible impact area of the epidemic was also wider than the areas with reported cases. The main driving factors of the scrub typhus epidemic in Zhejiang included endemic components such as natural factors, but there was significant heterogeneity in the composition of driving factors in different regions. Some regions were driven by spatiotemporal spread across regions, and the time autoregressive effect in individual regions could not be ignored. These results that monitoring of cases, vectors, and pathogens of scrub typhus should be strengthened. Furthermore, each region should take targeted prevention and control measures based on the main driving factors of the local epidemic to improve the accuracy of prevention and control.

## Background

Scrub typhus, also known as tsutsugamushi disease, is an acute natural focus disease caused by infection with *Orientia tsutsugamushi (Ot)*, which is transmitted through the bite of chigger mite larvae (1, 2). The typical clinical manifestations of the disease include high fever, severe headache, lymph node enlargement, eschar, and rash. In severe cases, scrub typhus can even lead to multiple organ failure and death (3, 4). Scrub typhus is currently an important Rickettsia disease worldwide, with approximately 1 million people becoming sick each year and over 1 billion people at risk of infection (5). The disease is prevalent mainly in Asia, Australia, the Indian Ocean, and the Pacific Islands (the Tsutsugamushi Triangle). However, in recent years, the epidemic area of scrub typhus has continuously expanded from the traditional Tsugamushi Triangle to include Africa, South America, Europe, and the Middle East (1, 6). China is one of the main endemic areas of scrub typhus. With the continuous discovery and reporting of endemic foci, scrub typhus has spread from southern to northern China, and the numbers of reported cases and affected counties have rapidly increased (7, 8). During 1952–1989, the incidence rate of scrub typhus in China remained low, with an average annual incidence rate of 0.13/100,000. Since 2006, the annual incidence rate of scrub typhus in China has increased exponentially, with the annual incidence rate increasing from 0.09/100,000 in 2006 to 1.6/100,000 in 2016, an increase of more than 16-fold (9, 10). Zhejiang Province is located in the coastal area of southeastern China and has a subtropical monsoon climate that is very suitable for the growth and reproduction of tsutsugamushi. According to the literature, Zhejiang Province is also considered a key region of scrub typhus, as it belongs to one of the four spatiotemporal clusters of scrub typhus in China (11).

In numerous previous studies, there have been detailed reports on various aspects, including the discovery history of scrub typhus, the species and ecological characteristics of Chinese chigger mites, the geographical distribution and seasonal fluctuations of different chigger mites, the time, region, and population distribution characteristics of the disease, and the ecological and social factors that affect the occurrence of scrub typhus (6, 12–15). However, some epidemiological issues related to scrub typhus are not yet fully understood. First, although the Chinese mainland has implemented direct network reporting of infectious diseases since 2004, thus far, only 41 kinds of infectious diseases have been legally reported; this list of diseases does not scrub typhus. Moreover, due to the high incidence of scrub typhus in rural areas, disease monitoring in rural areas, which can feature poor hygiene conditions, is generally weak. For example, due to the difficulty in detecting disease-characteristic scabs during examinations, scrub typhus is often misdiagnosed; thus, its incidence is highly likely to be underestimated (16, 17). Therefore, the disease characteristics obtained from CDCPIS may be incomplete. Second, influenced by different natural and social environments, there is significant heterogeneity in the incidence of diseases between different regions. However, relatively little research has been conducted on the driving factors of this disease in various regions at small scales. Therefore, this study employed the kriging interpolation method to estimate the possible incidence of scrub typhus in Zhejiang Province, China. Additionally, a multivariate time series model was applied to identify the main driving components affecting the occurrence of scrub typhus in different regions of Zhejiang to further provide supplementary evidence for understanding the characteristics of this disease.

## Materials and methods

### Setting and study area

This was an ecological study on scrub typhus in Zhejiang Province, a provincial-level administrative region of the People’s Republic of China; Hangzhou is its capital city. Zhejiang Province is located on the southeast coast of China on the southern wing of the Yangtze River Delta. The province has 11 prefecture-level cities, and the permanent population of Zhejiang Province is expected to reach 65.77 million by the end of 2022. In Zhejiang Province, mountainous areas account for 74.6%, water surfaces account for 5.1%, and flat areas account for 20.3%. Zhejiang Province is located in the central subtropical region and has a humid monsoon climate.

### Data collection

Data on scrub typhus patients diagnosed at medical institutions between January 2016 and September 2023 were collected from the China Disease Control and Prevention Information System (CDCPIS). The population data at the county level (90 counties in total) were exported from the system each year. The statistical data on the temporal trends, regional distributions, and demographics of patients with this disease can be automatically calculated and exported from the system. The diagnosis of scrub typhus is based on the key points of diagnosis and treatment techniques for scrub typhus (trial). On the basis of clinical diagnosis of cases, one of the following conditions can be met to confirm the diagnosis, including positive indirect immunofluorescence test (double serum IgG antibody titer increased by 4 times or more), positive PCR nucleic acid test, or isolation of pathogen.

### Ordinary kriging interpolation

Like traditional epidemiology, sampling methods are also commonly used in spatial epidemiology research. Through spatial interpolation analysis, researchers can use limited sample point data to estimate the values of all point positions on the map plane to better understand the spatial distribution of diseases. Krige proposed the kriging interpolation method in 1951, which has been mathematically proven to be a locally optimal linear unbiased estimation technique for spatial distribution data. Linear refers to the linear combination of sample values, unbiased refers to the mathematical expectation that the estimated value is equal to the theoretical value, and optimal refers to the minimum variance of the estimated error (18). The key step of the kriging method is to calculate the semivariance and then estimate the values of unmeasured points based on adjacent sample values (19). The parameters of the semivariance function and the nugget effect can be estimated by an empirical semivariance function:


(1) $$\gamma \left(h\right)=\frac{1}{2N\left(h\right)}\overset{N\left(h\right)}{\underset{i=1}{\sum }}{\left[Z_{obs}\left(x_{i}+h_{x},y_{i}+h_{y}\right)-Z_{obs}\left(x_{i},y_{i}\right)\right]}^{2}$$

In Equation (1) $\gamma \left(h\right)$ is the semivariance value at distance interval h, $N\left(h\right)$ is the number of sample pairs within distance interval h, and $Z_{obs}\left(x_{i}+h_{x},y_{i}+h_{y}\right)$ and $Z_{obs}\left(x_{i},y_{i}\right)$ are sample values at two points separated by distance interval h (20). The fitted semivariance function models usually include exponential, spherical, Gaussian, and circular models. In the model, the ratio of the partial sill (R_1_) to the sill value (R_0_ + R_1_) reflects the strength of the spatial correlation. The larger the value is, the stronger the spatial correlation. The ratio of the nugget value (R_0_) to the sill value (R_0_ + R_1_) reflects the magnitude of sample variability caused by random factors. The larger the value is, the greater the spatial variability caused by random effects. The closer the Mean Fitting Errors are to 0, and the smaller the Root Mean Square Errors are, the better the fitting effect is.

### Multivariate time series model

The spatiotemporal multicomponent model is based on measuring the Poisson branching process of population migration while incorporating seasonal effects, long-term trends and over-discretization characteristics. This model is widely used in multiregional time series data analysis. Assuming that the research area is divided into *I* blocks, $Y_{i,t}$ is the case count in region i=1,…,I at time t=1,…,T . The count $Y_{i,t}$ is formally assumed to follow a negative binomial distribution $\frac{Y_{\mathrm{it}}}{Y_{i,t-1}}\sim \mathrm{NegBin}\left(\mu_{\mathrm{it}},\psi \right)$ i=1,…,I,t=1,…,T , with an additively decomposed mean.


(2) $$\mu_{\mathrm{it}}=\nu_{\mathrm{it}}e_{\mathrm{it}}+\lambda_{\mathrm{it}}Y_{i,t-1}+\phi_{\mathrm{it}}\underset{j\neq i}{\sum }\omega_{ji}Y_{j,t-1}$$

In Equation (2) ψ is an overdispersion parameter such that the conditional variance of $Y_{\mathrm{it}}$ is $\mu_{\mathrm{it}}\left(1+\Psi \mu_{\mathrm{it}}\right)$ . The Poisson distribution results in a special case if ψ=0 . The first component $\nu_{\mathrm{it}}e_{\mathrm{it}}$ represents the endemic risk, which captures factors such as population, sociodemographic variables, long-term trends, seasonality, and the climate within the local area y. The endemic mean is proportional to an offset of known expected counts $e_{\mathrm{it}}$ , typically reflecting the population at risk. As a district-specific measure of disease incidence, the population fraction $e_{\mathrm{it}}$ is included as a multiplicative offset. Here, the population at the county level was incorporated into the endemic component as a multiplicative offset. The next two components were observation-driven epidemic components. The second component $\lambda_{\mathrm{it}}Y_{i,t-1}$ is the time autocorrelation risk, which measures the impact of past epidemics on the current incidence of infectious diseases. The third component $\phi_{\mathrm{it}}\underset{j\neq i}{\sum }\omega_{ji}Y_{j,t-1}$ denotes the spatiotemporal characteristics capturing the transmission from other counties. Each parameter $\nu_{\mathrm{it}}$ , $\lambda_{\mathrm{it}}$ , and $\phi_{\mathrm{it}}$ is a linear predictor of the form.


(3) $$\log\left(\cdot \mathrm{it}\right)=\alpha^{\left(\cdot \right)}+b_{i}^{\left(\cdot \right)}+\beta^{{\left(\cdot \right)}^{T}}z_{\mathrm{it}}^{\left(\cdot \right)}$$

In Equation (3) “.” is one of ν,λ,ϕ ; $\alpha^{\left(\text{.}\right)}$ indicates intercepts; and $b_{i}^{\left(\cdot \right)}$ denotes the random effects, which account for heterogeneity between districts. $z_{\mathrm{it}}^{\left(\cdot \right)}$ denotes exogenous covariates, including time effects, and $\beta^{{\left(.\right)}^{T}}$ denotes the coefficient of $z_{\mathrm{it}}^{\left(\cdot \right)}$ .

$\omega_{ji}$ is the spatial contiguity weight matrix, which describes the strength of transmission from region j to region i . There are usually three models of neighborhood weights, including the first-order neighborhood model, the power law model and the second-order neighborhood model. The variance components are estimated by maximizing the approximated marginal likelihood obtained via Laplace’s approximation. The optimal model was selected through the Akaike information criterion (AIC) for the model without random effects, and the score rules, such as the logarithmic score (“logs”), the ranked probability score (“rps”), and the Dawid–Sebastiani score (“dss”), were applied to compare the random effect models. The smaller the value is, the better the fitting effect of the model (21–23).

### Wavelet analysis

In the 1980s, Professor Morlet proposed wavelet analysis with a time–frequency multiresolution function, which can clearly reveal multiple change periods hidden in time series, fully reflect the change trends of the system at different time scales, and qualitatively estimate the future development trend of the system. The basic idea of wavelet analysis is to represent or approximate a signal or function using a family of wavelet functions. Therefore, wavelet functions are the key to wavelet analysis, referring to a type of function that has oscillation and can quickly decrease to zero. The wavelet function is $\psi \left(t\right)\in L^{2}\left(R\right)$ and satisfies in Equation (4):


(4) $$\overset{+\infty }{\underset{-\infty }{\int }}\psi \left(t\right)dt=0$$

The wavelet function $\psi \left(t\right)$ is called the basic wavelet, and the function family $\left\{\Psi_{a,b}\right\}$ is generated by the translation and expansion of $\psi \left(t\right)$ .


(5) $$\Psi_{a,b}=\frac{1}{\sqrt{a}}\Psi \left(\frac{t-b}{a}\right)\cdot a,b\in R,a\neq 0$$

In Equation (5), a is the scale factor, which is a coefficient concerning the scale, and b is a translation factor, which is a factor concerning the event (24, 25). This method is used to capture the periodic fluctuation pattern of scrub typhus.

### Statistical analysis

The multivariate time series model and wavelet analysis were run in R Studio (version 1.2.5001). ArcGIS software (version 10.1, SERI, Inc.; Redlands, CA, USA) was used for ordinary kriging interpolation. A *p* value less than 0.05 or a confidence interval (CI) that did not include 0 represented statistical significance for all the tests.

## Results

### Basic epidemiological characteristics of diseases

**Figure 1:**
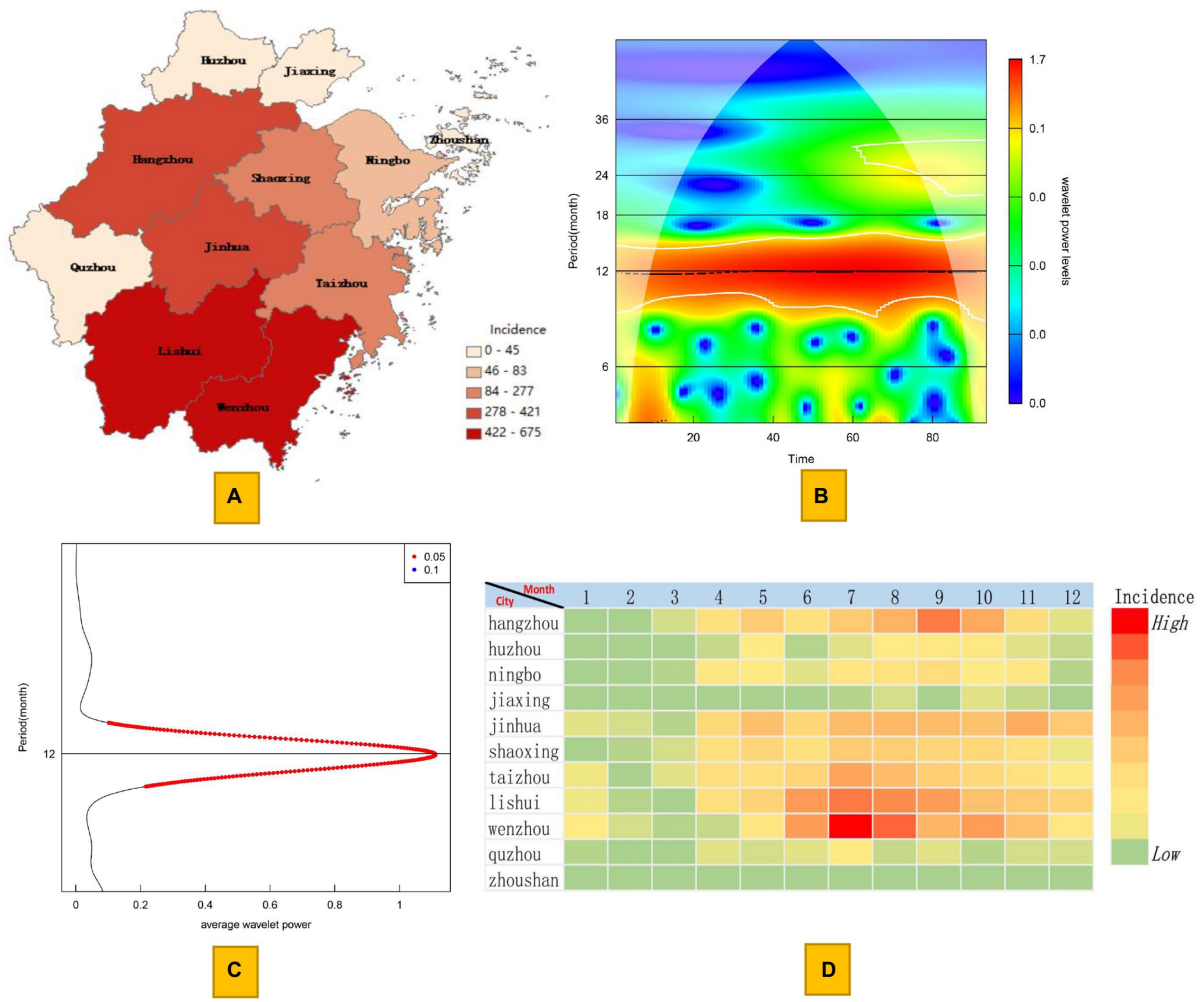
Distribution and seasonal characteristics of scrub typhus in Zhejiang Province. **(A)** Map of the city-level incidence. **(B)** Wavelet power spectrum of the monthly time series of scrub typhus from 2016 to 2023. **(C)** Average wavelet power spectrum of scrub typhus throughout the entire period. **(D)** Heatmap of the seasonal distribution of scrub typhus in 11 cities in Zhejiang Province.

From January 2016 to September 2023, 2,678 cases of scrub typhus were reported in Zhejiang Province, including 1 reported death, with an overall case fatality rate of 0.04%. From 2016 to 2022, the numbers of reported cases per year were 282, 294, 331, 372, 351, 482, and 260, respectively. As of September 2023, a total of 306 cases have been reported in the CDCPIS. Since 2016, the regions with the highest incidence have included Wenzhou city, Lishui city, Jinhua city, Hangzhou city, Taizhou city, and Shaoxing city, with 675, 551, 421, 380, 277, and 203 cases, respectively. Notably, there have been no reported cases in Zhoushan city, an island city located in the eastern part of Zhejiang Province. The cases in these six cities accounted for 93.61% of the total cases in the province (Figure 1A). Overall, the incidence of scrub typhus in Zhejiang Province exhibits obvious seasonal characteristics. Usually, the number of cases starts to increase in March and reaches its peak in July. Then, the incidence gradually decreases from August to November and decreases to a lower level in December. According to the results of the wavelet analysis, the seasonal characteristics of scrub typhus in Zhejiang Province exhibit a single annual peak, and stable seasonal fluctuations have been maintained over 12-month periods since 2016 (Figures 1B,C). However, the months of peak onset in different regions are not entirely consistent. Wenzhou city, Lishui city, Taizhou city, and Quzhou city are located in the southern region of Zhejiang Province. The peak incidence in these four cities occurs from June to August, accounting for 60.22% of the total number of cases throughout the year. Unlike the southern regions, Hangzhou, Huzhou, Jiaxing, and Ningbo, which are located in the northern part of Zhejiang Province, have relatively late onset peaks. The peaks in these four cities are concentrated from August to October, accounting for 61.58% of the total number of reported cases throughout the year. However, Jinhua city and Shaoxing city, which are located in the central region of Zhejiang Province, have not experienced particularly significant peak incidences, and the monthly incidences from May to November are relatively similar (Figure 1D).

Overall, the male-to-female ratio of patients with scrub typhus in Zhejiang Province was 0.86, which included 1,236 male patients and 1,442 female patients. The majority of cases of scrub typhus occur in the population aged 40 and above, with cases in the population aged 50–74 accounting for 73.45% of the total number of cases. There are 33 cases of children under 5 years old, accounting for 1.23%. The incidence rate in this age group ranges from 0.1cases/100,000 population to 0.3cases/100,000 population. In addition, 74.23% of the cases of scrub typhus affected farmers, which was much greater than the percentage of cases caused by other occupational types, followed by householders and unemployed workers, accounting for 7.73%.

### Kriging interpolation for estimating incidence at county level

First, commonly used circular semivariance functions, spherical semivariance functions, exponential semivariance functions, and Gaussian semivariance functions are used for selecting optimal models. The mean fitting errors of the four models mentioned above are −0.4428, −0.4221, −0.3537, and −0.4919, and the root mean squares of the fitting errors are 7.8098, 7.7246, 7.6214, and 8.0285, respectively. Based on the fitting error results, the exponential model with the smallest error should be selected. The nugget value of this model is 0, and the partial sill value is 57.1438. The ratio of the partial sill value to the sill value is 100%, indicating a strong spatial correlation of the data.

**Figure 2:**
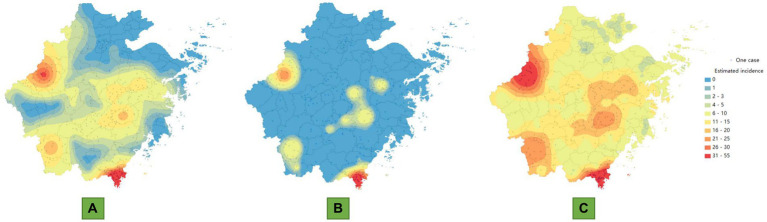
Maps of the estimated annual incidence of scrub typhus based on kriging interpolation. **(A)** Map of the mean estimated incidence. **(B)** Map of the 95% confidence intervals for the lower limit of incidence. **(C)** Map of the 95% confidence intervals for the upper limit of incidence.

By comparing the map of the estimated mean incidence with the actual incidence distribution, we found that the estimated area with case occurrence is relatively wider. From the perspective of annually reported cases, there are 41 counties in Zhejiang Province with an annual reported case count of less than 1, while from the estimated annual case count, the number of counties with less than 1 case has decreased to 21. There are four high-risk areas for the endemic, including Cangnan and Longquan Counties in the southern part of Zhejiang Province, Chun’an County in the southwest, and Xianju County in the central region. With these four counties as the center, the disease has gradually decreased toward the surrounding areas. In particular, in southwestern regions with fewer reported cases, such as Quzhou city, the actual number of infections may be greater than the current reported number. However, the number of cases in the northeastern region of Zhejiang Province is indeed low (Figure 2A).

From the estimated 95% confidence lower limit incidence map, it can be seen that the average annual numbers of cases in Cangnan County, Chun’an County, Longquan County, Qingyuan County, Xianju County, Xinchang County, Dongyang County, Jinyun County, Liandu District, Pan’an County, and Pujiang County remain high. However, from the 95% confidence upper limit incidence map, in addition to the 11 counties mentioned above, scrub typhus may also be widely present in other regions, including areas in the northeastern region, where there are no or very few cases reported. Overall, except for the 11 counties mentioned above, the average annual number of cases in other regions fluctuates between 0 and 15, indicating that the endemic in most regions is characterized by sporadic cases. In addition, even from the estimated upper limit, the number of cases in the central urban area of Hangzhou city, Jiaxin city and Huzhou city is very low, with an average annual maximum of no more than 5 cases (Figures 2B,C).

### Multivariate time series analysis

The monthly data from 2016 to 2023 were used to construct the multivariate time series model. First, three basic models were established to compare their performance. These three models included the first-order neighborhood model, the power law model and the second-order neighborhood model, and the incidence was assumed to follow a negative binomial distribution. The AIC values of these three models were 9047.76, 8835.85 and 8923.92, which suggested that the model with the neighborhood weight regarded as the power law mode would be better for modeling. Second, we compared two models assuming that the incidence would follow negative binomial and Poisson distributions, with the power law neighborhood regarded as the neighborhood weight. The AIC values of these two models were 8835.85 and 10,151.47, respectively, which suggested that the negative binomial distribution was better. Third, we considered random effects in the model. The proper scoring rules of the power law model without random effects were 0.4928 (logs), 0.2170 (rps), −0.4987 (dss) and 0.8655 (ses), while the indices of the power law model with random effects were 0.4241 (logs), 0.1849 (rps), −1.7655 (dss) and 0.6538 (ses). Therefore, the final model selected was the power law neighborhood model with random effects based on the assessment of the different models.

The estimated random effect variance parameters $\sigma_{\lambda }^{2}$ , $\sigma_{\phi }^{2}$ , and $\sigma_{\nu }^{2}$ were 0.48, 1.03 and 3.48, respectively, which indicated the heterogeneity of scrub typhus incidence among the different counties. The autoregressive component ( $\sigma_{\lambda }^{2}$ ) among districts showed little variation. The value of the endemic component ( $\sigma_{\nu }^{2}$ ) reached a maximum, which suggested that there was obvious spatial heterogeneity in the endemic component. The next most important component was the spatiotemporal component ( $\sigma_{\phi }^{2}$ ), which indicated that there was moderate variation between counties in terms of the effect attributed to the spatiotemporal component.

**Figure 3:**
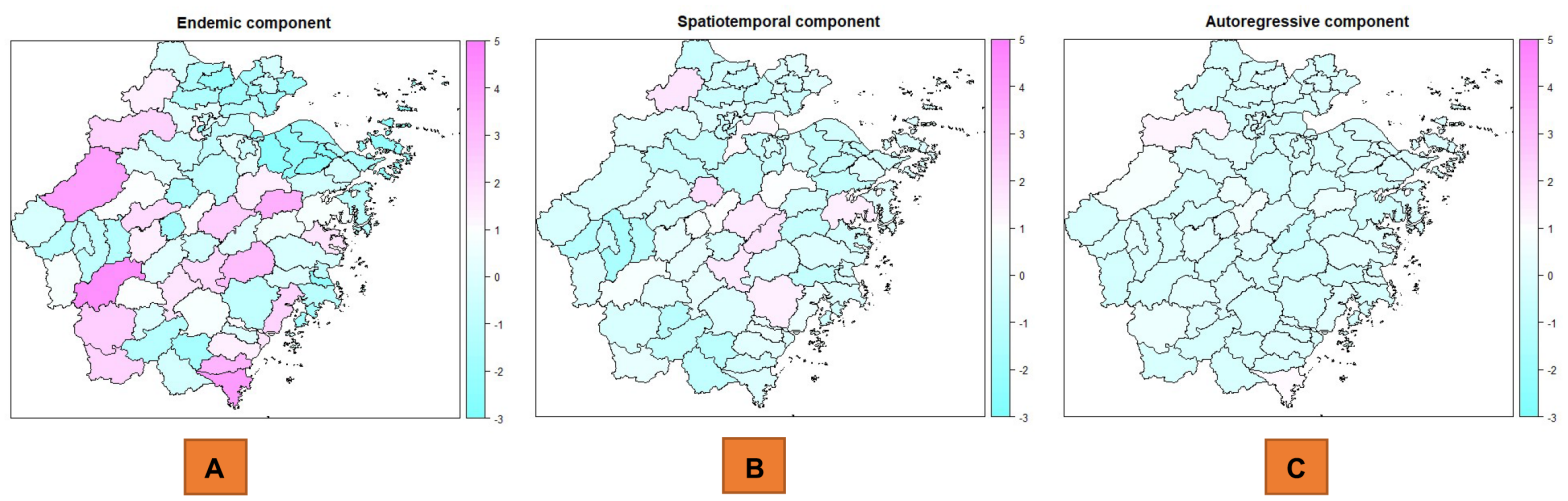
Random effects components of scrub typhus in Zhejiang Province, China, 2016–2023. **(A)** Endemic component at the county level. **(B)** Spatiotemporal component at the county level. **(C)** Autoregressive component at the county level.

There was obvious heterogeneity across counties for the mean values of the endemic component from the maps (Figure 3A). The spatiotemporal component also exhibited a certain degree of regional heterogeneity, while the autoregressive component, apart from making a significant contribution in a few regions, exhibited relatively homogeneous features in most regions. The endemic component values of the top 10 counties were Shuichang, Cangnan, Chun’an, Xinchang, Pingyang, Xianju, Longquan, Dongyang, Yueqing and Qingyuan. Interestingly, these areas were essentially consistent with the areas with high incidence risk. The top 10 counties with mean spatiotemporal component values greater than 0.9 were mostly located in the central parts of Zhejiang Province, near the regions with high incidence risk (Figure 3B). These counties included Pujiang, Pan’an, Dongyang, Jinyun, Yongjia, Yiwu and Shengzhou. The other three counties, Anji, Xiaoshan, and Ninghai, were relatively far from the high-risk areas. The autoregressive components of most counties were less than 0.8, and the counties with higher values mainly included areas such as Lin’an, Cangnan, and Chun’an (Figure 3C). Chun’an and Cangnan are high incidence incidence areas in Zhejiang Province, with one area in the north and one in the south, while Lin’an is one of the areas closest to Chun’an (Table 1).

**Table 1:** Counties with the top 10 highest values of the three driving factors.

County	Endemic component	County	Spatiotemporal component	County	Autoregressive component
Shuichang	4.40	Pujiang	1.91	Lin’an	1.28
Cangnan	4.13	Anji	1.81	Cangnan	1.10
Chun’an	3.99	Pan’an	1.81	Chun’an	0.89
Xinchang	3.45	Dongyang	1.70	Yiwu	0.72
Pingyang	3.23	Jinyun	1.63	Pujiang	0.54
Xianju	2.98	Ninghai	1.53	Longquan	0.48
Longquan	2.48	Yongjia	1.45	Xinchang	0.43
Dongyang	2.43	Xiaoshan	1.21	Luqiao	0.23
Yueqing	2.33	Yiwu	1.03	Sanmen	0.23
Qingyuan	2.28	Shengzhou	0.94	Fuyang	0.22

**Figure 4:**
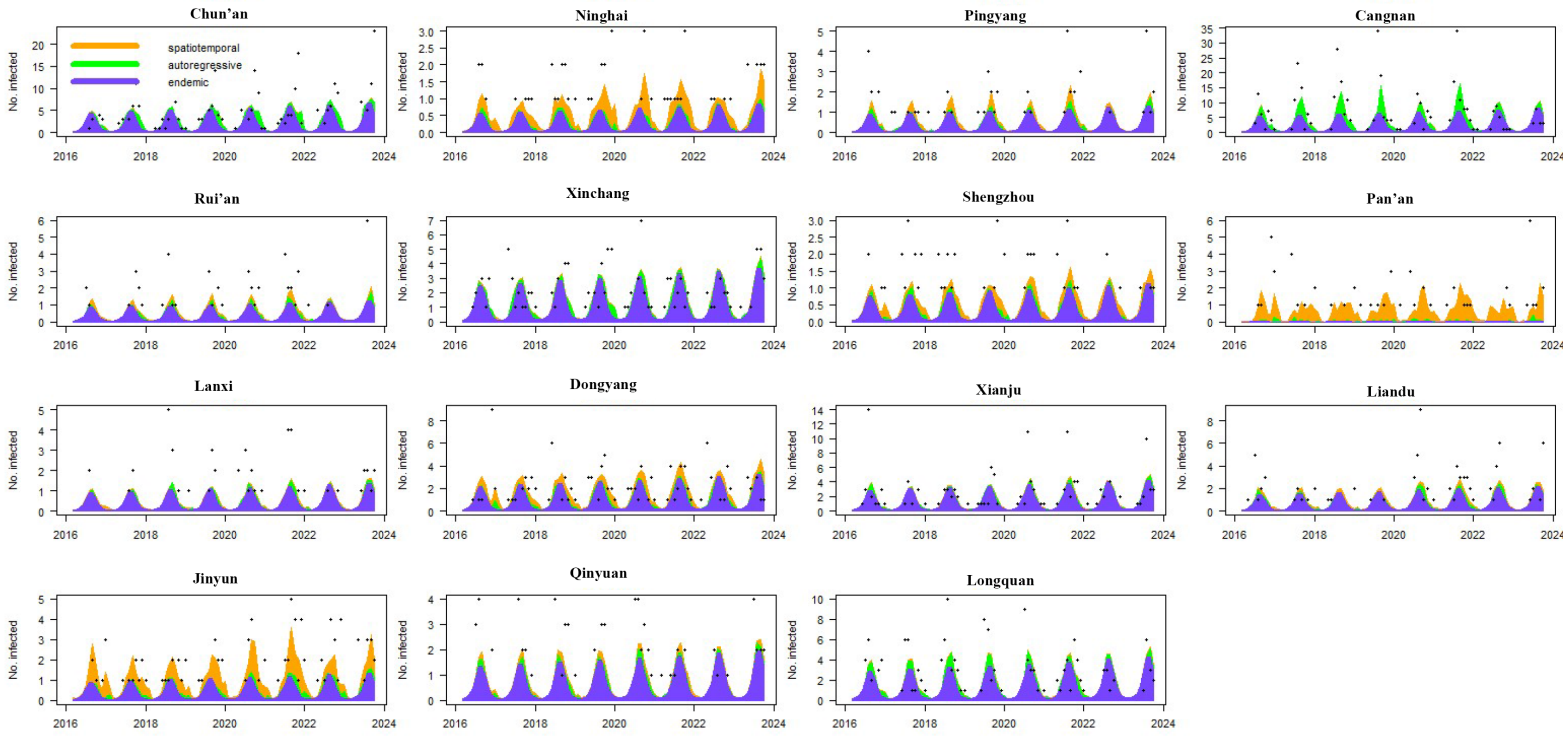
Fitted components in the multivariate time series model for the 15 counties with the highest case numbers. The black dots represent the monthly incidence counts, the blue area shows the endemic component, the green area shows the autoregressive component, and the orange area corresponds to the spatiotemporal component.

To identify the time-varying importance of the three components in the high-incidence areas (i.e., the areas with annual average case numbers greater than 50), the components were plotted along with the observed counts. The contributions of the three components in driving the endemic of scrub typhus over time and the seasonal characteristics were then visualized (Figure 4).

Among these 15 high-incidence counties, except for Pan’an County, the main driving factor for the endemic in other counties was the endemic component. The endemic situations in these 14 counties showed obvious seasonal characteristics, and all had a high incidence of a single peak within the year. For Pan’an County, the disease was almost always driven by spatiotemporal components, and this characteristic was maintained from 2016 to 2023. In addition, spatiotemporal components also made significant contributions in Ninghai, Jinyun, Pingyang, Dongyang, and Shengzhou and exhibited sustained impact characteristics. For Cangnan, Chun’an, Xinchang, and Longquan, which had the highest incidence of the disease, the autoregressive component also had a significant impact, especially in Cangnan County, where the contribution of the autoregressive component to the disease was almost one-fifth (Figure 4).

## Discussion

Similar to the findings of previous studies, the incidence of scrub typhus in Zhejiang Province exhibits obvious seasonal characteristics (9, 11). However, the timing and periodicity of this seasonal peak are different from those of the epidemics in other regions. For example, Fujian Province, which is adjacent to Zhejiang Province, has experienced a double peak of scrub typhus outbreaks within a single year (13). There are differences in the peak onset time among different countries. A study in Japan revealed two peaks in the incidence of scrub typhus, with a large peak in November and a smaller peak in May (26). Lewis reported that the incidence in the Maldives is concentrated in the summer (27). Studies on the incidence characteristics of scrub typhus in mainland China have revealed that China’s scrub typhus can be divided into summer type and autumn and winter type with 31° north latitude as the dividing line and single peak type and double peak type with 105° east longitude as the dividing line. In China, the area south of 31° N latitude and east of 105° E longitude has a bimodal summer pattern, with the main peak appearing in June or July and the second peak appearing in September or October, mainly covering Fujian, Guangdong, Guangxi, and other regions. The area south of 31° N latitude and west of 105° E longitude has a unimodal summer pattern, with the highest incidence occurring in July or August, mainly covering Sichuan, Yunnan, and other regions. The area north of 31° N latitude has unimodal autumn and winter patterns, with the peak occurring from September to December, mainly covering Shandong, Anhui, Jiangsu and other regions (11). In our study, we also found that there were significant differences in the timing of the seasonal peak of scrub typhus incidence among different cities in Zhejiang Province. Although the endemic generally exhibited a single peak in summer at the whole-province level, the peak of the incidence in the southern region of Zhejiang Province appeared earlier, while the peak of the incidence in the northern region appeared later. Moreover, there seem to be seasonal characteristics similar to those of transitional regions in the central region of Zhejiang Province. This seasonal difference is likely related to the distribution of chigger mites. *Leptotrombidium deliense* is the main carrier of scrub typhus in vast areas of southern China, and the peak population of this organism often occurs in summer. The hot and humid climate in summer is suitable for the growth, development, reproduction, and activity of mites (28). Second, other chigger mites, including *L. insulare*, *L. rubellum*, *L. kaohuense*, and *L. jishoum,* are also widely distributed. Climate conditions are also important factors affecting seasonal changes. In the Northern Hemisphere, the temperatures in spring and summer gradually advance from south to north, resulting in the emergence of suitable temperatures in the southern region to facilitate vector reproduction, leading to the first incidence peak in the southern region, followed by an increase in the incidence in the northern region.

In our study, only one case of death was reported, with a crude case fatality rate of 0.04%, which was lower than the national average (9). Among the reasons for the lower mortality rate are the high urbanization rate and gross domestic product (GDP) *per capita* in Zhejiang Province (21). Another reason may be that since 2006, China has once again included scrub typhus in the disease information reporting system for monitoring, and medical institutions have improved diagnostic sensitivity and treatment plans, such as the introduction of antirickettsial drugs (28, 29). However, as the symptoms of scrub typhus are similar to those of typhus, leptospirosis, and other diseases, it is easy to misdiagnose and miss the optimal diagnosis and treatment opportunity windows. Furthermore, delayed diagnosis can increase the risk of death by more than twofold. Therefore, the serious nature of the potential consequences of this disease should not be underestimated. The population distribution characteristics of scrub typhus in Zhejiang Province are essentially consistent with those of other studies, with cases occurring mainly among women and middle-aged and older adult farmers (30). The main reason for the high incidence of scrub typhus in some populations can be attributed to increased exposure opportunities. The active period of chigger mites is during the busy farming season, and the chances of exposure to rodents and chigger mites by field workers (i.e., mainly farmers) are much greater than those of other populations. Second, with the advancement of urbanization in China, a large number of middle-aged men have flooded into modern cities, with the result that older adult people have become the main force of agricultural production. Therefore, older adult people are more likely to be exposed to animal vectors during agricultural activities, resulting in an increased risk of infection (31, 32). In our study, there are few cases of child scrub typhus in Zhejiang Province, and the incidence rate is also significantly lower than that reported in Yunnan, Sichuan and other provinces (9). The possible reason for this situation is that people have a strong awareness of protecting children from insect bites, which reduces their exposure opportunities.The incidence of disease among women is greater than that among men, which may be related to women being the main rural residents in China at present (30).

Through kriging interpolation analysis, we roughly determined the possible distribution and severity of scrub typhus in Zhejiang Province and identified the hot and cold spots of the endemic. The southern, central, and western regions of Zhejiang Province each feature some endemic hotspots, and the occurrence in these areas is stable and consistent with the number of reported cases. In addition to these hotspots, we also found that the tsutsugamushi epidemic may be widespread, which is not reflected in the existing reported case data. Scrub typhus, a natural focal disease, is influenced by both natural and social factors. In terms of natural factors, the regional distribution of scrub typhus is closely related to natural geographical features such as meteorological factors, vegetation landscapes, animals/rodents which harbors the mites and topography. As the main carrier of scrub typhus in Zhejiang Province, the most suitable temperature range for *Leptotrombidium deliense* survival and reproduction is 18 ~ 28°C, and the most suitable relative humidity range is 95% ~ 100%. Scrub typhus can also survive in grasslands, shrubs, seawater, well water, and farmland ditch water (14). The host range of chigger mites is very wide, including mammals, birds, reptiles, amphibians, and even some arthropods. Rodents and some small mammals are the most common hosts of chigger mites (33). Zhejiang Province is mainly characterized by hilly terrain, with suitable temperature and humidity, wide vegetation coverage, and rich biodiversity, all of which are very suitable for the survival and reproduction of transmission vectors and rodents. Therefore, this study provides an important ecological foundation for the widespread occurrence of scrub typhus in Zhejiang Province. It should also be noted that the growth of the urban population is accompanied by changes in socioeconomic conditions, which can affect the incidence trend of scrub typhus. Excessive greenhouse gas emissions and environmentally unfriendly behaviors such as alterations in land use and animal population changes caused by human activities have exacerbated global warming, leading to changes in the host’s living environment and flora distribution. The implementation of ecologically friendly policies such as reducing industrial land, restoring forest wetlands, and prohibiting the burning of rice straw in rural areas has provided a favorable environment for the reproduction of rodents and mites, promoting the formation of new natural foci of scrub typhus. In addition, environmental hygiene conditions such as the cultivation of vegetable fields, maintenance of ditches and ponds near residences, raising poultry, and stacking weeds in front and behind residences also affect the occurrence of scrub typhus (34). The data produced in this study indicate that the central urban area in the northern region of Zhejiang Province is a stable cold spot. This may be related to the improvement of the urban hygiene environment and the promotion of healthy lifestyles among residents, which can reduce exposure opportunities for residents and decrease the incidence of infection.

Although the overall natural geographical environmental characteristics of Zhejiang Province are relatively similar and the scrub typhus epidemic may be widespread, multicomponent analysis revealed that the main factors driving the development of the disease vary greatly in different regions. The endemic component is undoubtedly the main driving factor for the disease, especially in areas with high numbers of reported cases, where its contribution is very prominent. This further confirms that there may be very suitable conditions for the survival of chigger mites in these areas. In addition to the impact of epidemiological factors, we believe that differences in diagnostic sensitivity and reporting standards should be attributed to endemic components. As previously estimated by interpolation, the actual area and scale of the endemic may be greater than the current levels reported by hospitals. Therefore, some areas with relatively low contributions of endemic components may not fully reflect the true incidence. Indeed, the low reported incidence of the disease in these areas could be due to inappropriate natural and social conditions and may also be due to reporting bias caused by the lack of timely and accurate diagnosis.

The spread of infectious diseases caused by personnel mobility is also another important factor in disease development. With the rapid development of the economy and improvements in transportation convenience, the cross-regional spread of infectious diseases requires increasing attention. In our research, we found that the surrounding areas of counties with higher local risk components in the central region exhibited significant cross-regional disease input characteristics. The other two counties far from the high-risk areas, Anji and Ninghai, also had significant spatiotemporal transmission components, indicating that the cases in these two regions may have been caused by the export of cases from farther endemic areas. Although the overall contribution of autoregressive components was not significant, the contribution of such factors is worthy of attention in three high-risk counties, namely, Chun’an, Lin’an, and Cangnan. Scrub typhus should be a seasonal epidemic, but the driving factors in these three regions suggest that there may be prolonged transmission of the disease in these counties. The reason for this difference may be the different compositions of the chigger mite species, such as the presence of *L. scutellare*, which is similar to that in Fujian Province and has led to the continuation of the endemic in winter (13). This hypothesis still lacks sufficient monitoring data for chigger mites, but it is worth conducting necessary investigations on this vector to gain a deeper understanding of chigger mites’ host selection, habitat characteristics, altitude distribution characteristics, and other issues.

From the perspective of the disease-driving factors in high-incidence counties, the main driving factor in most regions is the endemic component, which features obvious seasonal characteristics. This suggests that health education should be carried out for people in these high-risk areas, especially those with greater exposure opportunities, such as older adult farmers, to enhance their understanding of the causes, transmission routes, and symptoms of scrub typhus and to strengthen self-protection measures during high-risk periods of the endemic. For counties such as Pan’an, Ninghai, and Jinyun, the incidence is also affected by the output of cases from surrounding areas, especially in Pan’an County. The main factor driving the development of the incidence in this area is the impact of the surrounding endemic. The possible reason for this pattern is cross-regional labor mobility, i.e., short-term agricultural work such as tea picking and fruit picking in the surrounding areas during the busy agricultural season, which leads to the importation of infected cases. Therefore, for areas at high risk of spatiotemporal transmission, health education should be strengthened for key occupational groups to avoid going to high-risk areas as much as possible and to implement self-protection measures. Once suspicious symptoms appear, these people can seek medical attention in a timely manner to reduce the possibility of severe illness.

Several limitations should be noted in our study. First, the data for this study come from passive monitoring systems, and scrub typhus is not a legally reported infectious disease. Therefore, there may be some bias in the epidemiological characteristics presented in the reported data. Second, the disease may also be misdiagnosed, as it can be confused with leptospirosis, typhus and other diseases. There are no data to support the diagnostic coincidence rate, but it can be supplemented in subsequent research to obtain more accurate incidence data. Third, as the distribution of chigger mites and meteorological and geographical environment covariate data were not included in the multicomponent model, there may also be some bias in the contribution of each component, which needs to be further supplemented and improved in future research.

In conclusion, the seasonal patterns and demographic characteristics of scrub typhus in Zhejiang Province are essentially consistent with previous domestic research results. The case fatality rate of diseases is lower than the average level reported for the Chinese mainland. However, the actual incidence level may be higher than the current reported number of cases, and the possible impact area of the endemic is also wider than the areas with reported cases. Notably, the hot and cold spots of the endemic have been clearly identified. In addition, the main driving factors of the scrub typhus in Zhejiang are endemic components such as natural factors, but there is significant heterogeneity in the composition of driving factors in different regions. Some regions are driven by spatiotemporal spread across regions, and the time autoregressive effect in individual regions cannot be ignored. These findings suggest that monitoring of cases, vectors, and pathogens of scrub typhus should be strengthened, and the impact of human development and the modification of nature on the activities of vectors and host animals, as well as the spread of the endemic, should be further evaluated. Furthermore, each region should take targeted prevention and control measures based on the main driving factors of the local endemic to improve the accuracy of prevention and control.

## Data Availability

The original contributions presented in the study are included in the article/supplementary material, further inquiries can be directed to the corresponding authors.

## References

[1] Xu G Walker DH Jupiter D Melby PC. Arcari CM.A review of the global epidemiology of scrub typhus. PLoS Negl Trop Dis. (2017) 11:e0006062. doi: 10.1371/journal.pntd.0006062 29099844 PMC5687757

[2] Lee SU. Epidemiologic characteristics of scrub typhus on Jeju Island. Epidemiol Health. (2017) 39:e2017039. doi: 10.4178/epih.e2017039 28823118 PMC5675983

[3] Musa TH Ahmad T Wana MN Li W Musa HH Sharun K. The epidemiology, diagnosis and management of scrub typhusdisease in China. Hum Vaccin Immunother. (2021) 17:3795–805. doi: 10.1080/21645515.2021.1934355 34124995 PMC8437466

[4] Xu P Mao GM Jiang HY Ren YT Wang Y Liang GW. Analysis of the epidemiological and clinical characteristics of 65 patients with scrub typhus on the east coast of China. Ann Palliat Med. (2021) 10:5694–705. doi: 10.21037/apm-21-1100 34107715

[5] John Antony Jude Prakash/Prakash JAJ. Scrub typhus: risks, diagnostic issues, and management challenges. Res Rep Trop Med. (2017) 8:73–83. doi: 10.2147/RRTM.S105602 30050348 PMC6038894

[6] Richards AL Jiang J. Scrub typhus: historic perspective and current status of the worldwide presence of Orientia species. Infect Dis. (2020) 5:6. doi: 10.3390/tropicalmed5020049, PMID: 32244598 PMC7344502

[7] Peng PY Xu L Wang GX He WY Yan TL Guo XG. Epidemiological characteristics and spatiotemporal patterns of scrub typhus in Yunnan Province from 2006 to 2017. Sci Rep. (2022) 12:2985. doi: 10.1038/s41598-022-07082-x 35194139 PMC8863789

[8] Xin HL Sun JL Yu JX Huang JL Chen QL Wang LP. Spatiotemporal and demographic characteristics of scrub typhus in Southwest China, 2006–2017: an analysis of population-based surveillance data[J]. Transbound Emerg Dis. (2020) 67:1585–94. doi: 10.1111/tbed.13492 31975551

[9] Li ZJ Xin HL Sun JL Lai SJ Zeng LJ Zheng CJ. Epidemiologic changes of scrub typhus in China, 1952-2016[J]. Emerg Infect Dis. (2020) 26:1091–101. doi: 10.3201/eid2606.191168 32441637 PMC7258452

[10] Yao HW Wang YX Mi XM Sun Y Liu K Li XL. The scrub typhus in mainland China: spatiotemporal expansion and risk prediction underpinned by complex factors. Emerg Microbes Infect. (2019) 8:909–19. doi: 10.1080/22221751.2019.1631719 31233387 PMC6598543

[11] Yue YJ Ren DS Liu XB Wang YJ Liu QY Li GC. Spatio-temporal patterns of scrub typhus in mainland China, 2006-2017[J]. PLoS Negl Trop Dis. (2019) 13:e0007916. doi: 10.1371/journal.pntd.0007916 31790406 PMC6917297

[12] Chen AX Tian C Yang X Cao G Lu XF. Research progress on the distribution of scrub typhus vector chigger mites in China. Chin J Hyg Insect Equip. (2022) 28:556–9. doi: 10.19821/j.1671-2781.2022.06.021

[13] Qian L Wang Y Wei X Liu P RJS M Qian Q. Epidemiological characteristics and spatiotemporal patterns of scrub typhus in Fujian province during 2012–2020. PLoS Negl Trop Dis. (2022) 16:e0010278. doi: 10.1371/journal.pntd.0010278 36174105 PMC9553047

[14] Han L Sun Z Li Z Zhang Y Tong S Qin T. Impacts of meteorological factors on the risk of scrub typhus in China, from 2006 to 2020: a multicenter retrospective study. Front Microbiol. (2023) 14:1118001. doi: 10.3389/fmicb.2023.1118001 36910234 PMC9996048

[15] Li X Wei XY Yin WW Soares RJ Magalhaes XYY Wen L. Using ecological niche modeling to predict the potential distribution of scrub typhus in Fujian Province, China. Parasit Vectors. (2023) 16:15. doi: 10.1186/s13071-023-05668-6 36721181 PMC9887782

[16] John R Varghese GM. Scrub typhus: a reemerging infection[J]. Curr Opin InfectDis. (2020) 33:365–71. doi: 10.1097/QCO.0000000000000664 32868511

[17] Gu XL Qi R Li WQ Jiao YJ Yu H Yu XJ. Misdiagnosis of scrub typhus as hemorrhagic fever with renal syndrome and potential co-infection of both diseases in patients in Shandong Province, China, 2013-2014. PLoS Negl Trop Dis. (2021) 15:e0009270. doi: 10.1371/journal.pntd.0009270 33784301 PMC8009391

[18] Qu LL Xiao HY Zheng NJ Zhang ZY Xu Y. Comparison of four methods for spatial interpolation of estimated atmospheric nitrogen deposition in South China. Environ Sci Pollut Res. (2017) 24:2578–88. doi: 10.1007/s11356-016-7995-0 27826827

[19] Fu CC Zhang HB Tu C Li LZ Luo YM. Geostatistical interpolation of available copper in orchard soil as influenced by planting duration. Environ Sci Pollut Res. (2018) 25:52–63. doi: 10.1007/s11356-016-7882-8 27798802

[20] Wu HC Wang XY Xue M Xue M Wu C Lu QB. Spatial characteristics and theepidemiology of human infections with avianinfluenza a(H7N9) virus in five waves from 2013 to 2017 in Zhejiang Province, China. PLoS One. (2017) 12:e0180763. doi: 10.1371/journal.pone.0180763 28750032 PMC5531501

[21] Wu HC Wang XY Xue M Wu C Lu QB Ding ZY. Spatial-temporal characteristics and the epidemiology of haemorrhagic fever with renal syndrome from 2007 to 2016 in Zhejiang Province, China. Sci Rep. (2018) 8:10244. doi: 10.1038/s41598-018-28610-8 29980717 PMC6035233

[22] Wu HC Wu C Lu QB Ding ZY Xue M Lin JF. Spatial-temporal characteristics of severe fever with thrombocytopenia syndrome and the relationship with meteorological factors from 2011 to 2018 in Zhejiang Province, China. PLoS Negl Trop Dis. (2020) 14:e0008186. doi: 10.1371/journal.pntd.0008186 32255791 PMC7164674

[23] Wu HC Xue M Wu C Lu QB Ding ZY Wang XX. Scaling law characteristics and spatiotemporal multicomponent analysis of syphilis from 2016 to 2022 in Zhejiang Province, China. Front Public Health. (2023) 11:1275551. doi: 10.3389/fpubh.2023.1275551 37965512 PMC10642232

[24] Xiao JP Liu T Lin HL Zhu GH Zeng WL Li X. Weather variables and the El Niño southern oscillation may drive the epidemics of dengue in Guangdong Province, China. Sci Total Environ. (2018) 624:926–34. doi: 10.1016/j.scitotenv.2017.12.200 29275255

[25] Wu HC Xue M Wu C Lu QB Ding ZY Wang XX. Trend of hand, foot, and mouth disease from 2010 to 2021 and estimation of the reduction in enterovirus 71 infection after vaccine use in Zhejiang Province, China. PLoS One. (2022) 17:e0274421. doi: 10.1371/journal.pone.0274421 36126038 PMC9488823

[26] Ogawa M Hagiwara T Kishimoto T Shiga S Yoshida Y Furuya Y. Scrub typhus in Japan: epidemiology and clinical features of cases reported in 1998. Am J Trop Med Hyg. (2002) 67:162–5. doi: 10.4269/ajtmh.2002.67.162 12389941

[27] Lewis MD Yousuf AA Lerdthusnee K Razee A Chandranoi K Jones JW. Scrub typhus reemergence in the Maldives[J]. Emerg Infect Dis. (2003) 9:1638–41. doi: 10.3201/eid0912.030212 14720413 PMC3034347

[28] Peng PY Xu L Wang GX He WY Yan TL Guo XG. Epidemiological characteristics and spatiotemporal distribution of scrub typhus in mainland China in 1952-1989 and 2006-2017. Chin J Zoonoses. (2022) 38:818–20. doi: 10.3969/j.issn.1002-2694.2022.00.110

[29] Fan MY Walker DH Yu SR Liu QH. Epidemiology andecology of rickettsial diseases in the People’s republic of China. Rev Infect Dis. (1987) 9:823–40. doi: 10.1093/clinids/9.4.823 3326129

[30] Zhang WY Wang LY Ding F Hu WB Soares Magalhaes RJ Sun HL. Scrub typhus in mainland China, 2006–2012: the need for targeted public health interventions. PLoS Negl Trop Dis. (2013) 7:e 2493. doi: 10.1371/journal.pntd.0002493 PMC3873277 24386495

[31] Kuo CC Lee PL Chen CH Wang HC. Surveillance of potential hosts and vectors of scrub typhus in Taiwan. Parasit Vectors. (2015) 8:611. doi: 10.1186/s13071-015-1221-7 26626287 PMC4666075

[32] Wei Y Huang Y Luo L Xiao X Liu L Yang Z. Rapid increase of scrub typhus: an epidemiology and spatial-temporal cluster analysis in Guangzhou City, southern China, 2006-2012. PLoS One. (2014) 9:e101976. doi: 10.1371/journal.pone.0101976 25006820 PMC4090214

[33] Ding F Jiang WL Guo XG Fan R Zhao CF Zhang ZW. Infestation and related ecology of chigger mites on the Asian house rat (*Rattus tanezumi*) in Yunnan Province, Southwest China. Korean J Parasitol. (2021) 59:377–92. doi: 10.3347/kjp.2021.59.4.377 34470089 PMC8413864

[34] McMahon BJ Morand S Gray JS. Ecosystem change and zoonoses in the Anthropocene. Zoonoses Public Health. (2018) 65:755–65. doi: 10.1111/zph.12489 30105852

